# Evaluation of Cell-Specific Epigenetic Age Acceleration in People With Multiple Sclerosis

**DOI:** 10.1212/WNL.0000000000207489

**Published:** 2023-08-15

**Authors:** Vicki Maltby, Alexandre Xavier, Ewoud Ewing, Maria-Pia Campagna, Sandeep Sampangi, Rodney J. Scott, Helmut Butzkueven, Vilija Jokubaitis, Lara Kular, Steffan Bos, Mark Slee, Ingrid A. van der Mei, Bruce V. Taylor, Anne-Louise Ponsonby, Maja Jagodic, Rodney Lea, Jeannette Lechner-Scott

**Affiliations:** From the School of Medicine and Public Health (V.M., R.L., J.L.-S.), University of Newcastle, University Drive, Callaghan; Immune Health Program (V.M., A.X., J.L.-S.), Hunter Medical Research Institute; Department of Neurology (V.M., J.L.-S.), John Hunter Hospital, New Lambton Heights; School of Biomedical Sciences and Pharmacy (A.X.), University of Newcastle, University Drive, Callaghan, Australia; Department of Clinical Neuroscience (E.E., L.K., M.J.), Karolinska Institutet, Center for Molecular Medicine, Karolinska University Hospital, Stockholm, Sweden; Department of Neuroscience (M.-P.C., S.S., H.B., V.J.), Central Clinical School, Monash University, Victoria; Division of Molecular Genetics (R.J.S.), Pathology North, John Hunter Hospital, New Lambton Heights; MSBase Foundation (H.B.), Melbourne, Australia; Institute of Clinical Medicine (S.B.), University of Oslo,; Department of Neurology (S.B.), Oslo University Hospital, Norway; Flinders University (M.S.), Adelaide; Menzies Institute for Medical Research (I.A.M., B.V.T.), University of Tasmania, Hobart; Florey Institute of Neuroscience and Mental Health (A.-L.P.), The University of Melbourne; Centre of Epidemiology and Biostatistics (A.-L.P.), School of Population and Global Health, University of Melbourne; Murdoch Children's Research Institute (A.-L.P.), Royal Children's Hospital, Melbourne; and Centre for Genomics and Personalized Health (R.L.), School of Biomedical Science, Queensland University of Technology, Kelvin Grove, Australia.

## Abstract

**Background and Objectives:**

In multiple sclerosis (MS), accelerated aging of the immune system (immunosenescence) may be associated with disease onset or drive progression. DNA methylation (DNAm) is an epigenetic factor that varies among lymphocyte subtypes, and cell-specific DNAm is associated with MS. DNAm varies across the life span and can be used to accurately estimate biological age acceleration, which has been linked to a range of morbidities. The objective of this study was to test for cell-specific epigenetic age acceleration (EAA) in people with MS.

**Methods:**

This was a case-control study of EAA using existing DNAm data from several independent previously published studies. Data were included if .idat files from Illumina 450K or EPIC arrays were available for both a case with MS and an age-matched and sex-matched control, from the same study. Multifactor statistical modeling was performed to assess the primary outcome of EAA. We explored the relationship of EAA and MS, including interaction terms to identify immune cell-specific effects. Cell-sorted DNA methylation data from 3 independent datasets were used to validate findings.

**Results:**

We used whole blood DNA methylation data from 583 cases with MS and 643 non-MS controls to calculate EAA using the GrimAge algorithm. The MS group exhibited an increased EAA compared with controls (approximately 9 mths, 95% CI 3.6–14.4), *p* = 0.001). Statistical deconvolution showed that EAA is associated with MS in a B cell–dependent manner (*β*_int_ = 1.7, 95% CI 0.3–2.8), *p* = 0.002), irrespective of B-cell proportions. Validation analysis using 3 independent datasets enriched for B cells showed an EAA increase of 5.1 years in cases with MS compared with that in controls (95% CI 2.8–7.4, *p* = 5.5 × 10^−5^). By comparison, there was no EAA difference in MS in a T cell–enriched dataset. We found that EAA was attributed to the DNAm surrogates for Beta-2-microglobulin (difference = 47,546, 95% CI 10,067–85,026; *p* = 7.2 × 10^−5^), and smoking pack-years (difference = 8.1, 95% CI 1.9–14.2, *p* = 0.002).

**Discussion:**

This study provides compelling evidence that B cells exhibit marked EAA in MS and supports the hypothesis that premature B-cell immune senescence plays a role in MS. Future MS studies should focus on age-related molecular mechanisms in B cells.

## Introduction

Aging is a major risk factor of many medical conditions. During the natural aging process, the immune system undergoes drastic changes in composition and functionality, termed “immune senescence.” This process results in reduced adaptive immune response, increased susceptibility to infections, increased inflammation, and increased nonorgan-specific autoantibody production (reviewed in ref. 1). Multiple Sclerosis (MS) is a complex disease of the CNS, which is influenced by both genetic and environmental factors. A hallmark of MS is the dysregulation of the immune system. It has been demonstrated that people with (pw) MS have a shorter life span and increased all-cause mortality compared with their healthy counterparts.^2,3^ It is therefore possible that pwMS exhibit premature aging of immune cells.

Epigenetics, such as DNA methylation (DNAm), refers to heritable but modifiable mechanisms of genetic regulation that represent an interface for environmental and genetic factors to influence the genome. DNA methylation refers to the addition of a methyl group to a DNA nucleotide (usually the cytosine of a cytosine-guanidine pairing [CpG]). We, and others, have demonstrated that there are differences in the global methylation profiles of immune cell subtypes of cases with MS compared with non-MS controls.^4-7^ Most differences were observed in CD19^+^ B cells and monocytes, with less occurring in T cells.^4,7-9^ Furthermore, there have been distinct differences observed due to treatment, particularly after dimethyl fumarate use in both CD4^+^ T cells and monocytes.^10,11^

DNAm also changes with the natural aging process. This has been exploited by age-predicting algorithms (“epigenetic clocks”), which use a specific set of CpGs to predict an individual's biological age.^12^ The first-generation clocks included the pan-tissue Horvath clock^13^ and blood-specific Hannum clock.^14^ The discrepancy (residual) between DNAm age and chronological age is determined by regressing the epigenetic age on chronological age and is termed epigenetic age acceleration (EAA). These first-generation clocks were limited in that they only predicted chronological age and had a weak association with clinical parameters. This led to the development of the second-generation clocks, including the PhenoAge clock,^15^ which incorporates DNAm-based clinical biomarkers of aging, and the newest clock, the GrimAge clock, which also encompasses DNAm-based surrogates of plasma protein levels associated with aging and DNAm-based surrogates for smoking history (smoking pack-years).^16^ Both clocks predict biological age based on DNA methylation, but recent evidence shows that GrimAge provides superior prediction of all-cause mortality relative to all other clocks.^17^

Given the link between MS and aging and the evidence of EAA in other neurologic conditions, we reasoned that EAA might occur in pwMS. A previous study in pwMS compared with non-MS controls demonstrated that pwMS have accelerated aging according to the PhenoAge clock, which was particularly evident in women.^18^ However, given the supposed superiority of the GrimAge clock, we wanted to investigate whether additional information about epigenetic aging in MS could be derived using a larger population of people with MS using the GrimAge calculator. Furthermore, we investigated EAA in MS in all 5 major immune cell subtypes to test for cell-specific aging effects.

## Methods

### Study Population

This is a multicenter case-control study of existing DNA methylation data either available from previous studies or from public data repositories. The DNA methylation data used for whole blood GrimAge analysis consisted of 583 cases and 643 controls, which were merged datasets from 3 independently collected studies (2 Australian and 1 Swedish)^19,20^ (Ref. 21; Xavier et al., submitted).

The first Australian dataset was composed of baseline DNAm data from The Australian Multicenter Study of Environment and Immune function (Ausimmune study).^19^ Cases with MS for this study were recruited from Australian MS specialist centers, were primarily treatment naïve (82% treatment naïve), had relapse-onset phenotypes, and were at their first clinical diagnosis of CNS demyelination during sampling (for full study details, see Lucas et al.^19^ They were included in this study if they had available EWAS data and had converted to clinically definite MS by the 10-year follow-up study. Non-MS controls were matched for age, sex, and region. Controls were selected from the Australian electoral roll and matched to cases by age, sex, and region.

The second Australian dataset consisted of prevalent cases with MS who were exclusively of relapse onset and were on a variety of treatments at sample collection^21^ (publicly available dataset from Gene Expression Omnibus [GEO] [GSE106648]). This group was selected from the MSBase registry based on a diagnosis of relapse-onset MS, European ethnicity, female sex, Australian, minimum 5 years of clinical follow-up, minimum 3 relapse-independent Expanded Disability Status Scale (EDSS) scores recorded, and available genotype and whole blood methylation data. For this study, all participants from the second Australian study were used. This case group did not have controls, so were age matched and sex matched with data from 102 non-MS controls from a publicly available dataset obtained through Epigenome Wide Association Study (EWAS) data hub.^22^ Non-MS controls were selected by age and sex. Both Australian cohorts had been profiled using the Illumina Infinium EPIC arrays. Healthy controls were selected from Epidemiologic investigation of Multiple Sclerosis (EIMS) and matched to cases on smoking status (ref).

The Swedish dataset is publicly available from GEO (GSE106648) and consisted of prevalent cases with MS who were primarily on treatment during blood sampling (66%), mostly relapsing-remitting phenotype (86%), and had been profiled using the Illumina 450K arrays.^20^

Individually isolated cell types consisted of data pooled from 3 independent studies (independent of each other and the whole blood studies) (Australia, Sweden, and Norway) from our previously isolated studies of CD4^+^ T cells^4,6,7,23^(GSE130030) and CD19^+^ B cells.^7,9^ The CD4^+^ T-cell dataset consisted of 75 cases and 85 controls. The CD19^+^ B cells contained a subset of the same participants as the CD4^+^ T-cell dataset, plus additional samples, and consisted of 35 cases and 29 controls, pooled from 2 independent studies. Details of participant demographics has been previously published,^4-6,23^ but were all relapse onset, on a variety of treatments and had varying disease length. A summary is provided in eTables 1 and 2 (links.lww.com/WNL/C918).

### DNA Methylation Analysis

Details of DNA methylation analysis are given elsewhere.^4-6^ In brief, bisulfite-converted DNA was amplified, fragmented, and hybridized to either Illumina Infinium HumanMethylation450 BeadChip (Swedish/Norwegian data from CD4^+^ T cells) or Infinium MethylationEPIC arrays (Australian data) using standard protocols.

Raw IDAT files were processed in R using the ChAMP R package.^24,25^ To summarize, IDAT files were loaded and filtered to remove badly performing probes, low-performing samples (with a detection *p* value >0.01), probes located on known sequence variations, and probes located on X and Y chromosomes. Beta values were then normalized using the BMIQ method.^26^ Batch effects on both array and chip levels were corrected using the Combat algorithm.^27^ Immune cell proportion (cell%) estimation was performed using the R package Epidish with the epidish function (doi.org/10.1186/s12859-017-1511-5), with the reference-based inference method *CIBERSORT* (doi.org/10.1038/nmeth.3337).

### Epigenetic Age Calculations and Statistical Analysis

AgeAccelGrim is defined as the residual resulting from regressing EpigeneticAge on the chronological age and was the primary test variable for this study and is henceforth referred to as the EAA.^13^ For this study, EAA was derived using the first-generation Horvath and Hannum clock algorithms and the second-generation PhenoAge and GrimAge clock algorithms, which have all been previously described.^13-16^ The GrimAge clock is constructed as a composite of DNA methylation-based markers for plasma proteins, which include adrenomedullin (ADM), beta-2-microglobulin (B2M), growth differentiation factor 15 (GDF-15), plasminogen activation inhibitor 1 (PAI-1), and tissue inhibitor metalloproteinase 1 (TIMP1) and smoking history (PACKYRS). These DNAm surrogates are indicated by the prefix DNAm (e.g., DNAmB2M).

To assess the effect of EAA on MS outcome, we performed logistic regression with MS as outcome and age, sex, and study group as independent variables. This model structure was also used to assess the association of the plasma proteins with MS. To investigate cell-specific signal from whole blood, we used the interaction term ***Cell***%× ***EAA*** within the following logistic model:
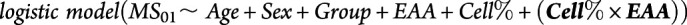


where MS_01_ represents the binary MS outcome (MS vs control), and *Age*, *Sex* and *Group* were used as covariates to correct for age, sex, and study of origin. We used the beta coefficient of the interaction term as the index of effect size and direction and *p* value for statistical significance to represent the cell-dependent EAA relationship to MS risk. In purified CD19^+^ B cells, we used Pearson correlation tests to relate the age-adjusted DNAm surrogate markers for both beta-2-microglobulin (B2M) and smoking pack-years to EAA.

### Standard Protocol Approvals, Registrations, and Patient Consents

Written and informed consent was obtained from all participants. Methods were conducted in accordance with institutional guidelines on human subject experiments. Data were shared through data transfer agreements, which were approved by the Hunter New England Local Heath District Human ethics committee (HNE-LHD HREC), the Karolinska Institute, Monash University, and the Australian National University. Data collected/generated in Australia were approved by the HNE-LHD HREC, approval numbers 2019/ETH12346 and 2020/STE05703. Data collected/generated in Sweden were approved by Regional Ethical Board, approval numbers 2004/1-4:6 and 2009/2107-31/2.

### Data Availability

Raw idat files for EWAS studies are available from Gene Expression Omnibus (GEO) (GSE106648) (Australia 2) and GEO (GSE106648, GSE130029) (Swedish). Other data not published in the article because of space limitation may be shared (anonymized) at the request of any qualified investigator to the corresponding author and will be subject to approval from the steering committee.

## Results

### EAA in the Whole Blood

**Figure 1:**
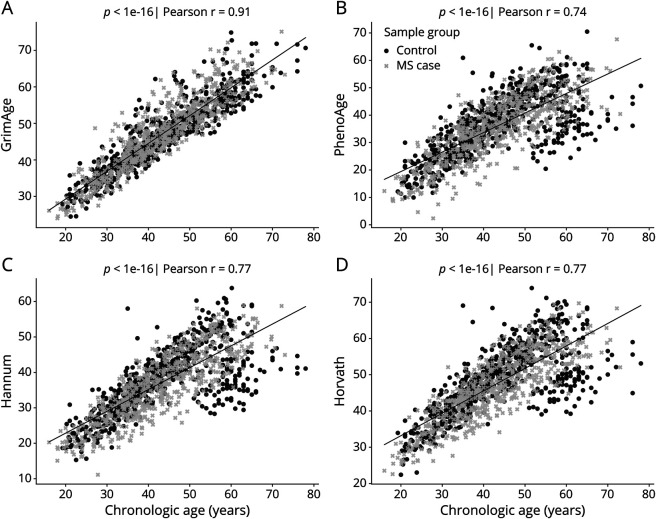
Correlation Between Chronological Age and GrimAge Epigenetic Clock Scatter plots showing correlation between chronological age and Grimage (A), phenoAge (B), Hannum (C), and Horwarth (D) clocks in whole blood analysis. Light gray = Case with MS. Black = Control. The black line represents the best-fit line.

We observed a strong correlation between chronological age and epigenetic age in the whole blood of 1226 individuals using all 4 clocks (R = 0.72 [PhenoAge], R = 0.77 [Hannum and Horvath, and *p* < 0.001]) (Figure 1, B–D). However, the correlation was the strongest using the GrimAge clock (R = 0.91, 95% CI 0.90–0.92, *p* < 1 × 10^−16^) (Figure 1A). Given the previously described superiority of the GrimAge clock^17^ in combination with our results, only the GrimAge clock was used in further analysis.

**Figure 2:**
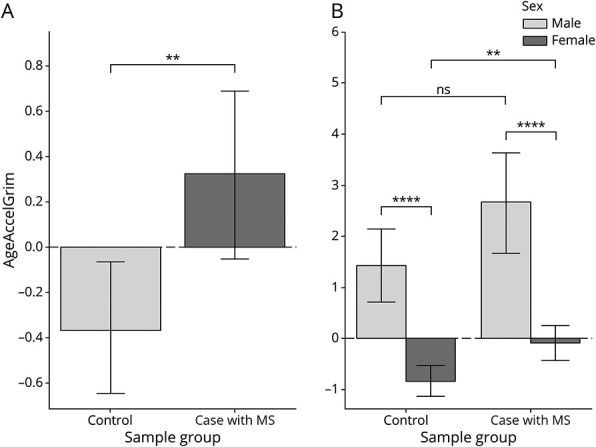
Cases With MS and Male Sex Have Increased GrimAge in Whole Blood Samples Bar graph showing cases (light gray) and controls (dark gray). (A) Whole blood comparison of EAA in cases and controls. (B) Whole blood comparison of EAA in male and female individuals in both case and control groups. Error bars represent the standard error of mean. **p < 0.01, ***p < 0.001, ****p < 0.0001. EAA = epigenetic age acceleration; ns = not significant.

Using the GrimAge clock, we calculated EAA and observed a statistically significant increase in the 583 cases compared with the 643 non-MS controls (Figure 2A), with the MS case group exhibiting an EAA approximately 9 months more accelerated than the control group (difference (mean case – mean control) = 9.0, 95% CI 3.63–14.36, *p* = 0.001). To assess for potential confounders in the data arising from either treatment use or study of origin, we calculated EAA separately for each study group and for participants who were not on treatment (N = 171, those who were on interferon beta (N = 32) and those on glatiramer acetate (N = 5) (eFigure 1, links.lww.com/WNL/C914). We find that treatment did not effect EAA (95% CI here) nor did study of origin (Australia 1 mean difference (case – control) 0.74, 95% CI 0.073–1.41; Australia 2 mean difference: 2.01, 95% CI 1.14–2.88, Swedish mean difference: 1.4, 95% CI 0.54–2.32; *p* > 0.05) with the exception that the Australia 2 study participants (both cases and controls) had a slightly lower EAA than the other 2 groups (difference −1.84; 95% CI −2.33 to −1.34) *p* = 5.8 × 10^−13^) (eFigure 2, links.lww.com/WNL/C915). In addition, we performed a correlation analysis between EAA and disease duration in the Swedish cohort only because the Australian cohorts were early-onset MS and found no correlation (R = 0.15; 95% CI -0.016 – 0.31; *p* = 0.075) (eFigure 3, links.lww.com/WNL/C916). Further analysis using a logistic model accounted for study of origin within the model.

Previous work demonstrates that male individuals have an increased EAA compared with female individuals.^28^ Therefore, we investigated the EAA in male and female individuals separately. We found in our cohort that female individuals also exhibit negative EAA (difference = −2.43, 95% CI −3.07 to −1.79, *p* = 1.10 × 10^−15^). However, we observed that female cases with MS displayed increased EAA relative to their non-MS counterparts (difference = 0.75, 95% CI 0.29–1.21, P_female individuals_ = 1.92 x 10^−3^), while male individuals with MS also indicated a trend toward increased EAA relative to male individuals without, but this did not reach statistical significance (difference = 1.29, 95% CI 0.072–2.51, P_male individuals_ = 0.052) (Figure 2B).

The GrimAge calculator uses DNAm surrogates for 7 plasma proteins related to biological age and a DNAm surrogate for smoking pack-years. To further understand the possible underlying biological contributors of EAA between cases and controls, we evaluated the relative signal from each of these DNAm-based plasma protein surrogates. We found that DNAmB2M (Beta-2-microglobulin) contributed significantly to the signal (difference = 10,189, 95% CI −8,015 to 28,394, *p* = 7.2 × 10^−5^), as did DNAmPACKYRS (smoking pack-years) (difference = 2.14, 95% CI 0.85–3.42, *p* = 0.002) (Table 1).

**Table 1:**
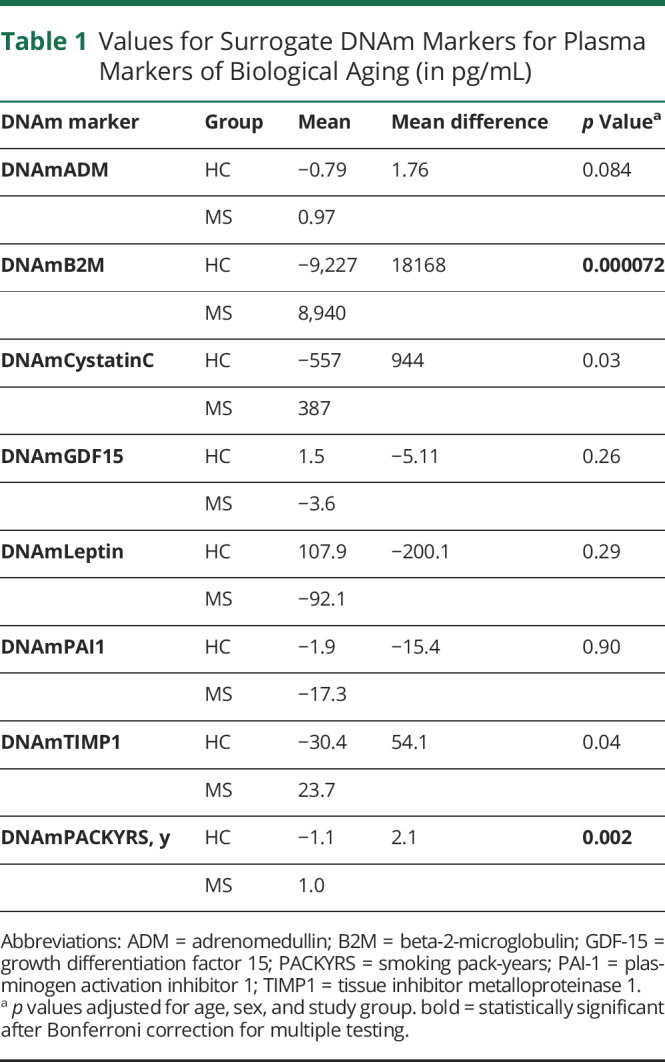
Values for Surrogate DNAm Markers for Plasma Markers of Biological Aging (in pg/mL)

### Immune Cell–Specific Analysis of EAA in MS

**Figure 3:**
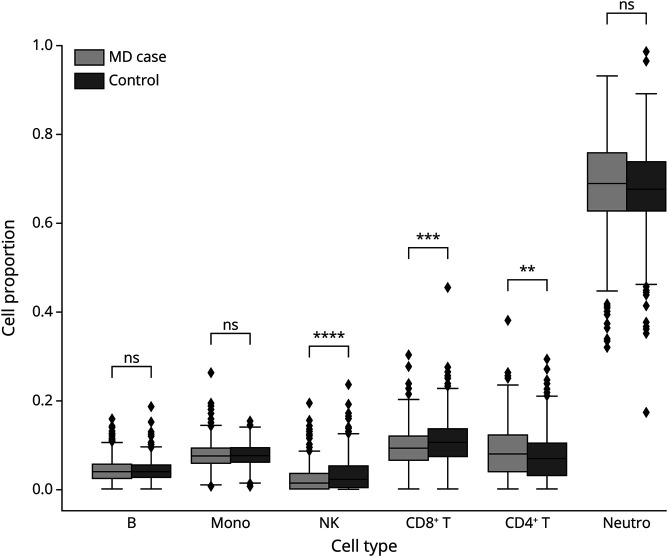
Cell Proportion Estimates From the Whole Blood Analysis Tukey Box Plot showing mean and interquartile range of cell proportions (… list all cell types in words…) in cases with MS (light gray) and controls (dark gray) in the merged dataset. Black diamonds indicate outliers. **p < 0.01, ***p < 0.001, ****p < 0.0001, ns = not significant.

Previous studies found that isolated cell types have varying levels of age acceleration.^18^ Therefore, the differences observed in EAA may be influenced by cellular heterogeneity in the whole blood samples. To test this hypothesis, we first used the R package *Epidish* to estimate cell proportions within the whole blood. We identified statistically significant differences between cases and controls in the proportion of natural killer (NK) cells (difference = −0.01, 95% CI −0.012 to −0.0062, *p* = 1.34 × 10^−08^), CD8^+^ T-cell proportions (difference = −0.011, 95% CI −0.016 to −0.0057, *p* = 5.92 × 10^−05^), CD4^+^ T-cell proportions (difference = 0.01, 95% CI 0.0037–0.016, *p* = 1.58 × 10^−03^), but no differences in B cells or monocytes (Figure 3).

**Figure 4:**
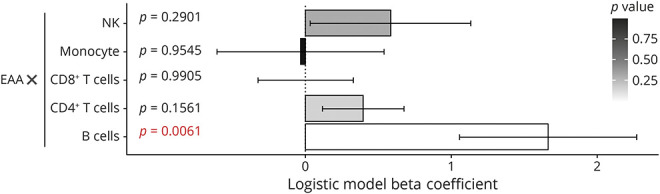
B Cells Demonstrate a Marked Effect on GrimAge Bar graphs show the Beta coefficient for each cell type interaction term of cell% x EAA. Shades of gray indicate *p* value (white = 0, black = 1). Error bars represent the standard error of mean. EAA = epigenetic age acceleration.

To assess whether the EAA difference in MS is acting in a cell-specific manner, we tested for interaction effects with cell% in the logistic regression model after accounting for age, sex, and study (Figure 4, eTable 3, links.lww.com/WNL/C918). Of interest, CD19^+^ B cells showed a statistically significant interaction effect on EAA (β_int_ = 1.7, 95% CI 0.30–2.78, *p* = 0.0061), whereas there was no significant interaction between EAA and CD4^+^ T cells, CD8^+^ T cells, NK cells, or monocytes (*p* > 0.05).

### EAA in Isolated CD4^+^ T Cells and CD19^+^ B Cells

**Figure 5:**
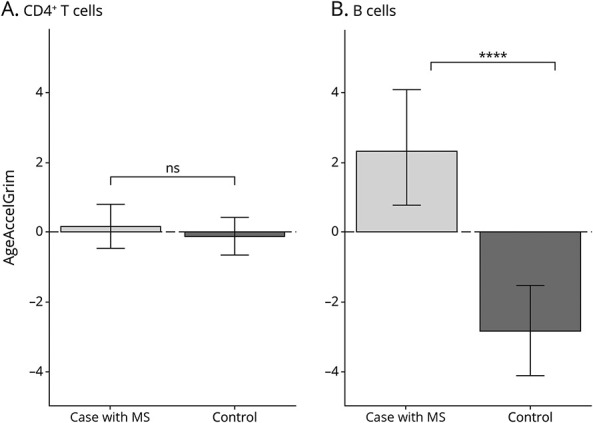
B Cells Have Increased EAA Bar graph showing the EAA for cases (light gray) and controls (dark gray) in isolated (A) CD4^+^ T cell or (B) CD19^+^ B-cell populations. Error bars represent the standard error of mean. EAA = epigenetic age acceleration.

We have previously assessed DNAm in isolated cell subtypes in pwMS and controls.^4,6-8^ To determine whether the cell-specific DNA methylation values predicted from the whole blood represent a true reflection of individual cell subtype, we validated our results by again using the GrimAge calculator to evaluate EAA on data from 3 independent studies of previously isolated CD4^+^ T cells^4,6,7^ and 2 studies of CD19^+^ B cells.^7,9^ As predicted from the whole blood results, CD4^+^ T cells showed no evidence of EAA (difference = 0.26, 95% CI −0.59–1.11, *p* = 0.55) (Figure 5A). Also consistent with results from the whole blood analysis, EAA in isolated CD19^+^ B cells in cases were approximately 5.1 years older than the controls (difference = 5.11, 95% CI 2.84–7.38, *p* = 3.18 × 10^−5^) (Figure 5B).

**Figure 6:**
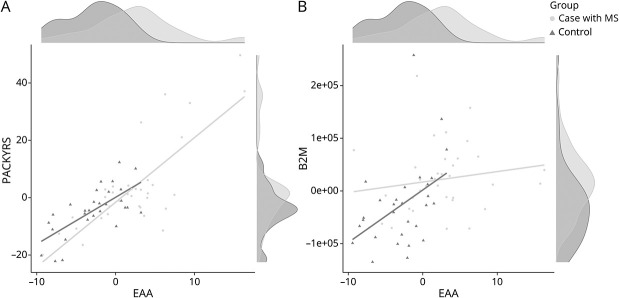
Correlation Between Isolated CD19+ B Cells, DNAmPACKYRS, and DNAmB2M Scatter plot showing correlation between (A) DNAmPACKYRS (smoking pack-years) (age adjusted) and EAA and (B) DNAmB2M (beta-2-microglobulin) (age adjusted) and EAA in cases with MS (black dots) and controls (gray triangles). Density plots along edge show distribution of signal for each of cases (light gray) and controls (dark gray). DNAm = DNA methylation; EAA = epigenetic age acceleration.

To evaluate whether the signal from the isolated CD19^+^ B cells was also primarily coming from DNAmB2M and DNAmPACKYRS, we evaluated the correlation between EAA and each of DNAmB2M and DNAmPACKYRS from the B-cell dataset (see eTable 2, links.lww.com/WNL/C918 for details on each dataset). There was a statistically significant correlation between EAA and both DNAmPACKYRS (R = 0.78, 95% CI 0.67–0.86, *p* = 1.86 × 10^−14^) and DNAmB2M (R = 0.37 95% CI 0.13–0.56, *p* = 2.89 × 10^−3^) (Figure 6 and eTable 3). Independent analysis of the dataset from each study shows no statistically significant differences between study groups (eFigure 4, links.lww.com/WNL/C917).

## Discussion

We have used the GrimAge clock to assess EAA (AgeAccelGrim) in pwMS and found a modest but statistically significant EAA in pwMS compared with that in non-MS controls. EAA seems to be most prominent in male individuals, driven primarily by CD19^+^ B cells rather than other cell types and by the DNAm surrogate markers for beta 2 microglobulin (B2M) and smoking pack-years (PACKYRS). We believe this study supports the concept of B-cell immune senescence as a contributor to MS disease.

In this study, we identified B cells as the primary contributors to EAA, from the 5 cell types examined. B-cell production has been shown to decrease in the bone marrow with increasing chronological age. A distinctive group of B cells has been characterized in animal studies, termed age-associated B cells (ABCs).^29,30^ In vitro and animal studies of ABCs suggest that they tend to secrete more proinflammatory cytokines,^29^ have longer, more stable interactions with T cells,^30,31^ and are more likely to secrete autoantibodies than younger B cells. Furthermore, B cells with similar phenotypic properties to ABCs appear in humans affected with several autoimmune diseases.^30,32,33^ Given the success of B-cell depleting therapies for MS is plausible that B-cell aging contributes to MS risk. Further studies characterizing the functional properties of B cells in MS will be of interest.

The GrimAge clock uses DNAm surrogate markers for various plasma proteins.^16^ We observed that DNAmB2M contributes significantly to EAA in both the whole blood and more specifically B cells. Beta-2-microglobulin is the light chain that is common to HLA-A, HLA-B, HLA-C, and most MHC antigens.^34^ It is involved in immune system function and has been shown to increase in the presence of cytokines such as IFN-gamma, and IL-6.^35^ The level of B2M has been proposed as a biomarker of multiple diseases, including but not limited to multiple myeloma,^36^ prostate cancer,^37^ and viral infection.^38^ Beta-2-microglobulin has been investigated as a potential biomarker of MS activity (reviewed in ref. 39); however, results were conflicting, and its reliability was questionable. It may, however, be a marker for efficacy of therapies with interferon beta and cladribine.^40^ Future studies that investigate changes in EAA over time in longitudinal cohorts with treatment data will be of interest to determine whether EAA slows with treatment use.

We also observed a strong correlation with the DNAm surrogate for smoking pack-years and EAA, both in the whole blood and B cells. Smoking has been shown to be associated with 7.91 years of GrimAge acceleration^16^ and is a leading factor for many age-related diseases. It is well established that smoking can alter DNAm patterns,^41-44^ and DNAmPACKYRS was found to be a better predictor of all-cause mortality than self-reported smoking.^16^ While the original Hannum and Horvath clocks required smoking to be included as a covariate, the GrimAge clock includes smoking as a predictor of mortality; therefore, it is expected to be influenced by smoking status.

A recent meta-analysis of biological, social, and environmental factors associated with epigenetic aging in blood identified male sex as consistently being associated with increased EAA, regardless of ethnicity.^28^ Our results are consistent with this, demonstrating that men have higher EAA than women, irrespective of disease status. Another study of EAA in MS^18^ used the Horvath, Hannum, and PhenoAge clocks to evaluate EAA in cases with MS compared with a control population. The Hannum and Horvath clocks are designed to predict chronological age.^13,14^ The PhenoAge and GrimAge clocks predict mortality and phenotypic age and use biological markers of aging in their predictions; therefore, only the PhenoAge clock is suitable for comparison with results from the GrimAge clock.^15,16^ When we compare results from the study conducted by Theodoropoulou et al., we find our results are consistent with their results from the PhenoAge clock, in that female individuals have lower EAA in general, but female individuals with MS have a higher EAA than their unaffected female counterparts. It is possible that parity may play a role in the reduced EAA seen in women. A recent study by Campagna et al.^45^ showed that women with MS with previous pregnancies had a lower EAA by 2.27 years when compared with women with MS who had never been pregnant. It will be of interest for future studies to include data on parity in women.

Given that the data for this study originate from multiple different study centers, there is potential for bias in the study results. However, isolated cells were processed in a similar manner between sites (PBMCs [peripheral blood mononuclear cells] were isolated by density gradient followed by magnetic bead separation).^4,6,7,9^ In the whole blood, the DNA samples from the Australian studies were processed at the same center, at the same time (Newcastle). Furthermore, we have previously demonstrated that methylation signals are robust, with sample storage and blood processing having negligible effects on methylation profiles.^46^ We also corrected for study of origin during our regression analysis and performed subgroup analysis for each study of origin and did not observe differences in the magnitude of EAA between studies. In addition, the Australia 1 cohort was comprised largely of participants at their first demyelinating event, whereas the Swedish cohort had varying disease length. We therefore used a correlation analysis between EAA and disease duration. This demonstrated no association of EAA with disease duration suggesting not only that disease duration did not significantly affect our results but also shows that EAA is present from early on in disease development.

This study is limited by its retrospective cross-sectional nature. As such, we were unable to adjust for all possible clinical variables that have been linked with epigenetic age acceleration such as life course psychosocial stressors,^47^ exposure to toxins,^48^ pregnancy,^49^ or comorbidities such as BMI, infection, cardiovascular disease, lung function, alcohol use, and mental health indices such as depression—all of which have been associated with GrimAge.^28^ Some of these data are missing from our datasets, and other data are not present in large enough numbers for subanalysis. We were also unable to assess the effect of progression on EAA because the number of participants who were either secondary or primary progressive were too small for subgroup analysis. Further studies focused on these populations will be of interest.

Many participants in this study were on treatment during sampling. We analysed the effect of treatment (treatment vs no treatment) in the 2 larger cohorts where this information was available and no differences between group were found (eFigure 1, links.lww.com/WNL/C914). This study was underpowered to detect differences at the isolated cell type level. Therefore, we find it unlikely that treatment has influenced our results. Larger prospectively collected studies that also investigate changes between newly diagnosed cases with MS and those with longer disease duration, particularly those on treatments, and include relevant clinical parameters such as parity, BMI, and comorbidities will be of interest in the future.

We have shown that B cells in MS exhibit a marked EAA. This is attributed to beta-2-microglobulin and smoking pack-years. These results support the hypothesis that premature B-cell immune senescence plays a role in MS.

## Glossary

ABCs: age-associated B cells
B2M: beta-2-microglobulin
CpG: cytosine-guanidine pairing
DNAm: DNA methylation
EAA: epigenetic age acceleration
EIMS: Epidemiologic investigation of Multiple Sclerosis
EWAS: Epigenome Wide Association Study
GEO: Gene Expression Omnibus
PACKYRS: smoking pack-years
